# Retained arterial catheter following patient self-discontinuation—a case report

**DOI:** 10.1093/jscr/rjad727

**Published:** 2024-01-16

**Authors:** Alexandria Byskosh, Kevin Olsen

**Affiliations:** St. Elizabeth’s Medical Center, Department of Surgery, Chobanian and Boston University’s Avedisian School of Medicine, Boston, MA 02135, United States; Department of Anesthesiology, Moffitt Cancer Center, Tampa, FL 33612, United States

**Keywords:** arterial line, retained line, vascular surgery

## Abstract

A patient self-discontinues arterial line resulting in retention of arterial catheter. The retained catheter was identified on bedside ultrasound and the patient required radial artery exploration and removal of catheter under general anesthesia. We describe potential contributing factors and solutions to catheter design and placement as well as next steps once a retained catheter has been identified.

## Introduction

Arterial catheters play a vital role in the hemodynamic monitoring of patients who often require monitoring in the perioperative period. Challenges exist in maintaining catheter sterility, security, and functionality, especially in patients with acute encephalopathy who often requirement restraint both chemically and physically. The methods used to secure the catheter, including adhesive bandages, tape and even suture, can compromise the integrity of the catheter and lead to complications. Strict adherence to manufacturer recommendation for securement and hospital protocols can help prevent catheter related mishaps. Written Health Insurance Portability and Accountability Act authorization has been obtained from the patient for this case report.

## Case description

**Figure 1:**
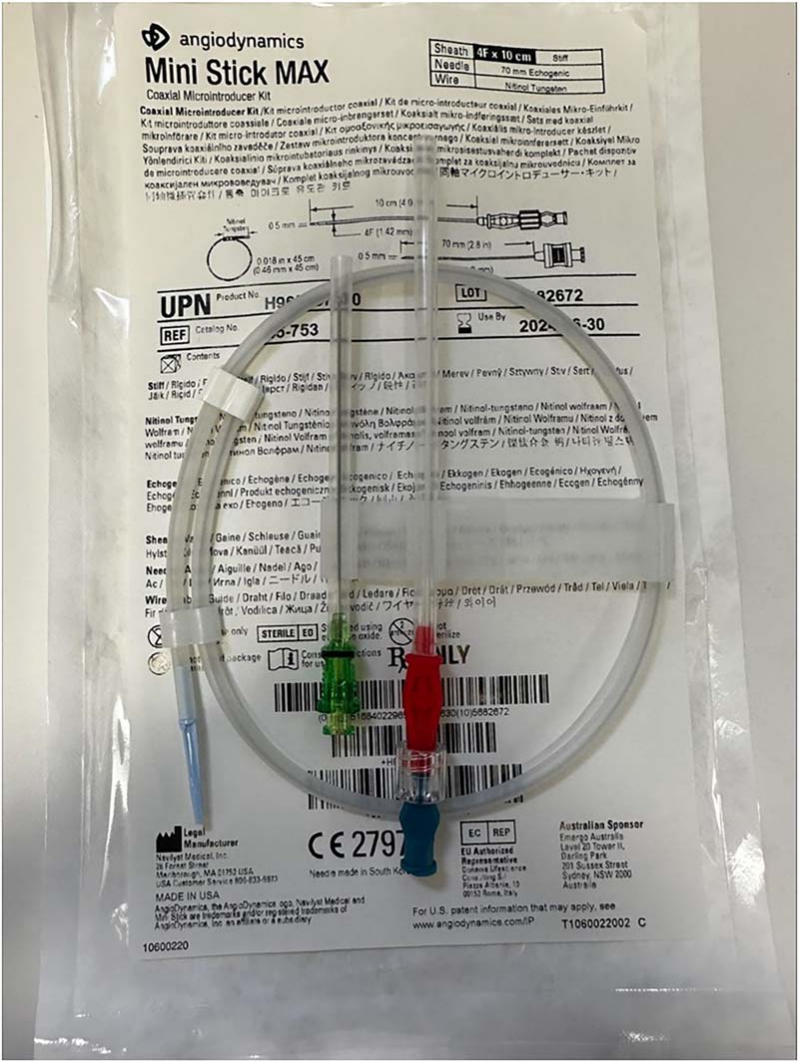
4F × 10 cm Mini Stick MAX arterial line insertion kit.

**Figure 2:**
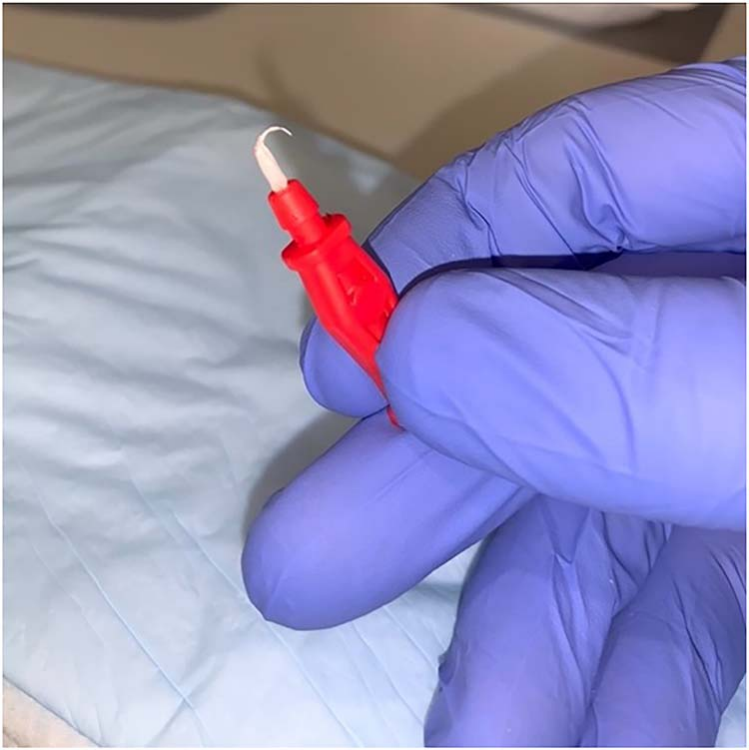
Catheter hub twisted off from the body of the catheter.

**Figure 3:**
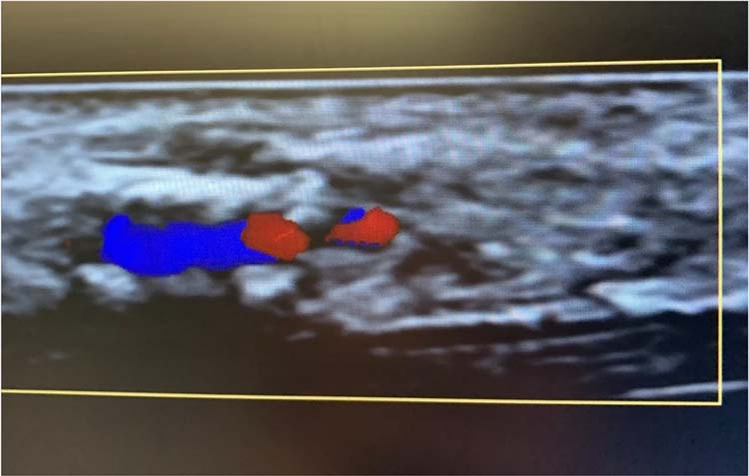
Color flow doppler distinguishing between arterial versus venous flow.

**Figure 4:**
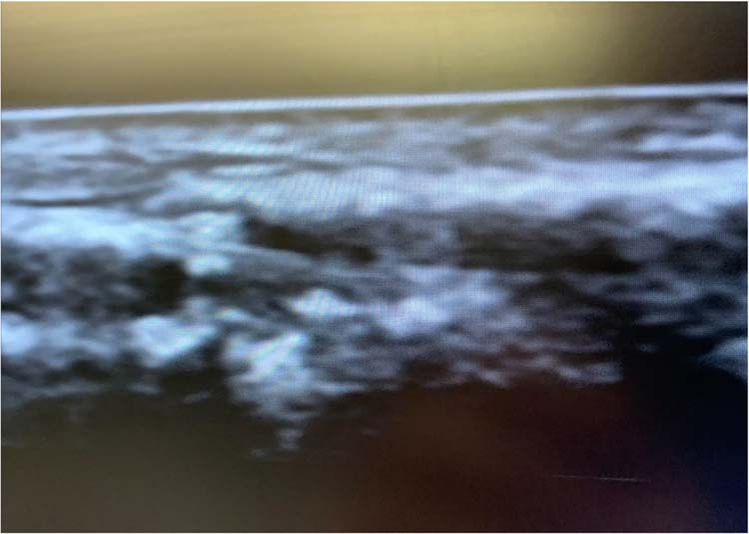
Non-color doppler showing retained arterial line.

**Figure 5:**
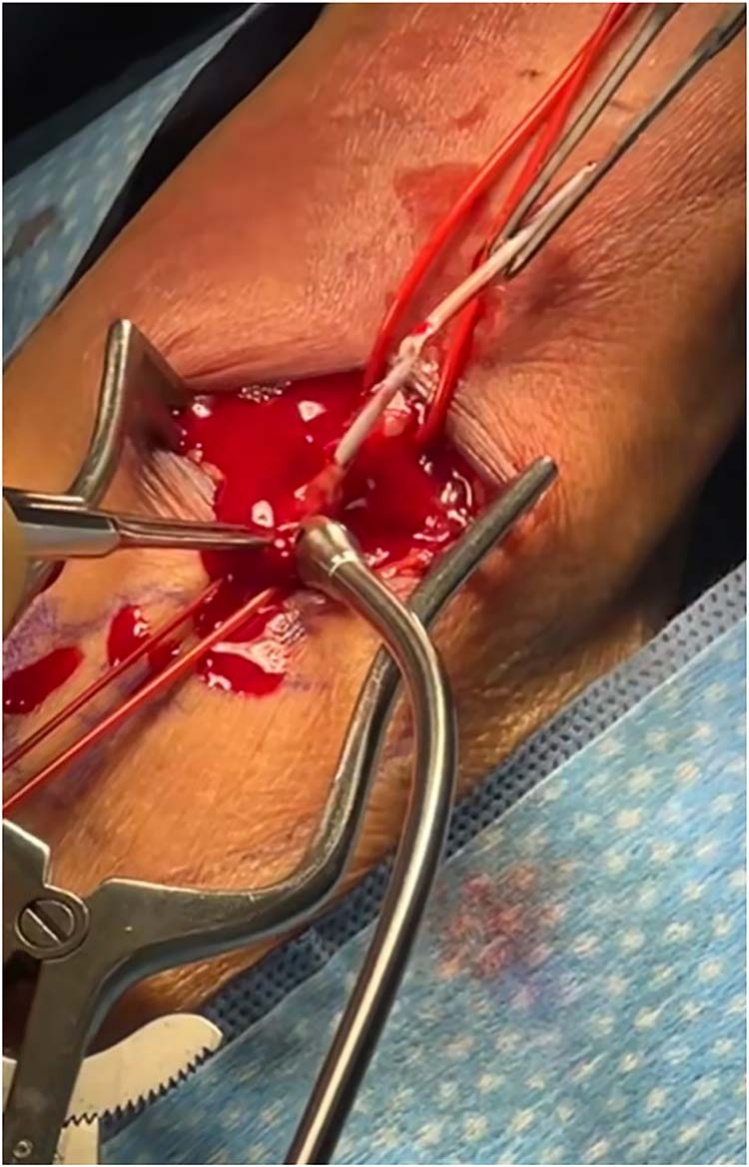
Intraoperative visualization of retained catheter with clot.

An 87-year-old male presented after a syncopal fall with a subdural hematoma who underwent emergent evacuation. Postoperatively he was admitted to the intensive care unit with a 4F × 10 cm Mini Stick MAX (Fig. 1) radial arterial line that was sutured in place. On postoperative day one, he became confused and self-discontinued the arterial line. Upon examination, it was noted that a portion of the catheter had twisted upon itself and the removed catheter was not completely intact (Fig. 2), indicating the possibility of a retained foreign body. A bedside ultrasound was used to identify and confirm the catheter still within the artery (Figs 3 and 4) and vascular surgery was consulted. The patient was brought to the operating room for a right radial artery exploration and removal of catheter under general anesthesia (Fig. 5). The patient recovered uneventfully with intact distal radial blood flow.

## Discussion

Arterial catheterization is an important procedure which allows for continuous hemodynamic monitoring as well as direct access for lab draws including arterial blood gas in critically ill patients. Retained catheters following removal are a rare, yet, morbid complication of arterial catheterization [1–3]. The exact mechanisms leading to catheter retention can be multifactorial and often involve device design, manipulation technique, securement technique, anatomical variation, or a combination of these factors.

**Figure 6:**
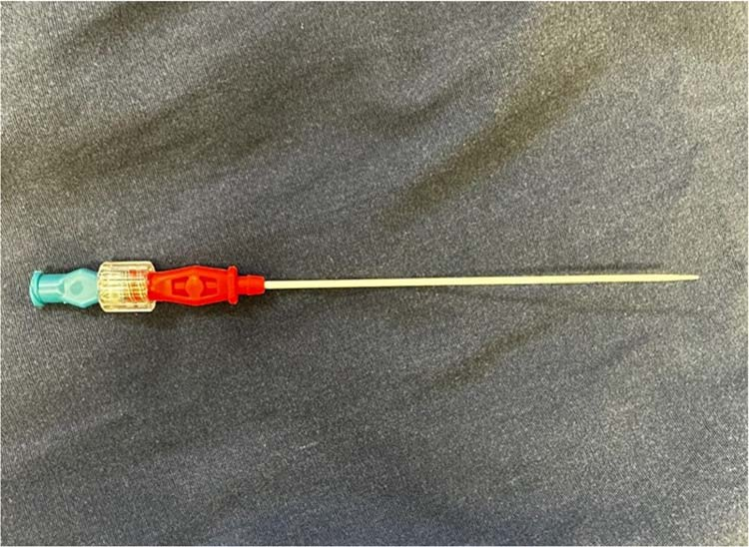
Outer catheter with a connection port and an inner dilating catheter of the coaxial system.

**Figure 7:**
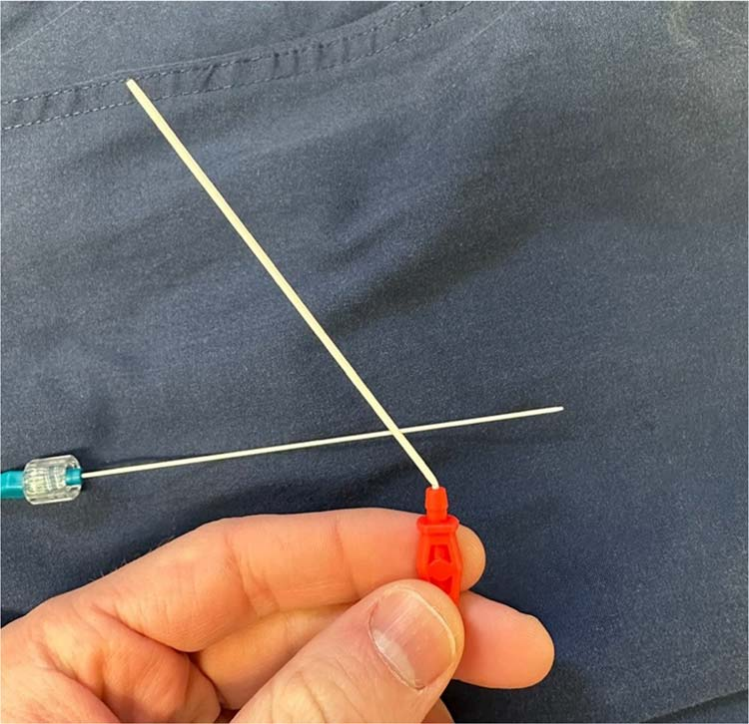
Kinked catheter once dilator is removed.

Regarding the Mini Stick Max coaxial micro-introducer kit, the specific design features of the device may contribute to the potential for catheter malfunction. The coaxial system consists of multiple components, including the outer catheter with a connection port and an inner dilating catheter, which are designed to be used together for precise catheter placement (Fig. 6). The inner dilator is used during insertion and should be removed prior to securing the catheter in place. The securement of the catheter is often done with suture around the red connector port but suture may slip down onto the white catheter itself, causing occlusion/kinking. Any deviation from the recommended technique or suboptimal device handling may lead to kinking (Fig. 7), increasing the risk of catheter retention.

Another factor that might contribute to catheter retention is the presence of anatomical variations or patient-specific factors. These can include tortuous or narrow vessels, anatomical anomalies, or adhesions, which may hinder the smooth removal of the catheter. Ultrasound-guided insertion is useful to identify potential challenges and plan the procedure accordingly. Additionally, ensuring proper training and expertise among proceduralists in handling such devices can help minimize the risk of complications.

The prompt recognition of retained catheter fragments is crucial to prevent potential complications. Once a retained catheter fragment is identified, the management approach should be individualized based on several factors, including the patient’s clinical condition, the characteristics of the retained segment, and the potential risks and benefits of removal.

Conservative management with close monitoring and serial imaging may be considered if the patient is stable, the retained fragment is small and asymptomatic, and the risk of attempting extraction outweighs the potential benefits. In some cases, such as the one described, surgical intervention or endovascular techniques may be necessary to remove the retained catheter segment, especially if it poses a significant risk of complications such as infection, thrombosis, or embolization.

To prevent retained catheter complications, a systematic approach should be followed during device insertion, manipulation, and removal. Proceduralists should adhere to standardized techniques and guidelines, maintain clear communication, and ensure proper training and competence in handling specific devices like the Mini Stick Max coaxial micro-introducer kit. Manufacturers should also provide comprehensive instructions for use, including clear guidance on device removal to minimize the risk of retention. A suggested improvement includes adding suture specific loops/holes on the lateral portions of the red connector port, similar to some central lines, so that suture remains in the correct place and cannot contribute to kinking of the catheter.
